# Narrative cave art in Indonesia by 51,200 years ago

**DOI:** 10.1038/s41586-024-07541-7

**Published:** 2024-07-03

**Authors:** Adhi Agus Oktaviana, Renaud Joannes-Boyau, Budianto Hakim, Basran Burhan, Ratno Sardi, Shinatria Adhityatama, Hamrullah, Iwan Sumantri, M. Tang, Rustan Lebe, Imran Ilyas, Abdullah Abbas, Andi Jusdi, Dewangga Eka Mahardian, Sofwan Noerwidi, Marlon N. R. Ririmasse, Irfan Mahmud, Akin Duli, Laode M. Aksa, David McGahan, Pindi Setiawan, Adam Brumm, Maxime Aubert

**Affiliations:** 1 School of Humanities, Languages and Social Science, Griffith University, Gold Coast, Queensland Australia; 2 Pusat Riset Arkeometri, Organisasi Riset Arkeologi, Bahasa, dan Sastra, Badan Riset dan Inovasi Nasional, Jakarta, Indonesia; 3 The Griffith Centre for Social and Cultural Research (GCSCR), Griffith University, Gold Coast, Queensland Australia; 4 Center for Prehistory and Austronesian Studies (CPAS), Jakarta, Indonesia; 5 Geoarchaeology and Archaeometry Research Group, Southern Cross University, Lismore, New South Wales Australia; 6 Pusat Riset Arkeologi Prasejarah dan Sejarah, Organisasi Riset Arkeologi, Bahasa, dan Sastra, Badan Riset dan Inovasi Nasional, Jakarta, Indonesia; 7 Pusat Kolaborasi Riset Arkeologi Sulawesi, Makassar, Indonesia; 8 Australian Research Centre for Human Evolution, Griffith University, Brisbane, Queensland Australia; 9 Korps Pecinta Alam, Universitas Hasanuddin, Makassar, Indonesia; 10 Departemen Arkeologi, Fakultas Ilmu Budaya, Universitas Hasanuddin, Makassar, Indonesia; 11 Balai Pelestarian Kebudayaan Wilayah XIX, Makassar, Indonesia; 12 Badan Layanan Umum Museum dan Cagar Budaya, Direktorat Jenderal Kebudayaan, Jakarta, Indonesia; 13 Pusat Riset Arkeologi Lingkungan, Maritim, dan Budaya Berkelanjutan, Organisasi Riset Arkeologi, Bahasa, dan Sastra, Badan Riset dan Inovasi Nasional, Jakarta, Indonesia; 14 KK Desain Komunikasi Visual, Fakultas Seni Rupa dan Desain, Institute Teknologi Bandung, Bandung, Indonesia; 15 School of Environment and Science, Griffith University, Queensland, Australia

**Keywords:** Archaeology, Cultural evolution

## Abstract

Previous dating research indicated that the Indonesian island of Sulawesi is host to some of the oldest known rock art^1–3^. That work was based on solution uranium-series (U-series) analysis of calcite deposits overlying rock art in the limestone caves of Maros-Pangkep, South Sulawesi^1–3^. Here we use a novel application of this approach—laser-ablation U-series imaging—to re-date some of the earliest cave art in this karst area and to determine the age of stylistically similar motifs at other Maros-Pangkep sites. This method provides enhanced spatial accuracy, resulting in older minimum ages for previously dated art. We show that a hunting scene from Leang Bulu’ Sipong 4, which was originally dated using the previous approach to a minimum of 43,900 thousand years ago (ka)^3^, has a minimum age of 50.2 ± 2.2 ka, and so is at least 4,040 years older than thought. Using the imaging approach, we also assign a minimum age of 53.5 ± 2.3 ka to a newly described cave art scene at Leang Karampuang. Painted at least 51,200 years ago, this narrative composition, which depicts human-like figures interacting with a pig, is now the earliest known surviving example of representational art, and visual storytelling, in the world^3^. Our findings show that figurative portrayals of anthropomorphic figures and animals have a deeper origin in the history of modern human (*Homo sapiens*) image-making than recognized to date, as does their representation in composed scenes.

## Abstract (web summary)

A cave art scene at Leang Karampuang, Indonesia, dated to at least 51,200 years ago using laser-ablation uranium-series imaging, depicts human-like figures interacting with a pig.

## Main

**Fig. 1:**
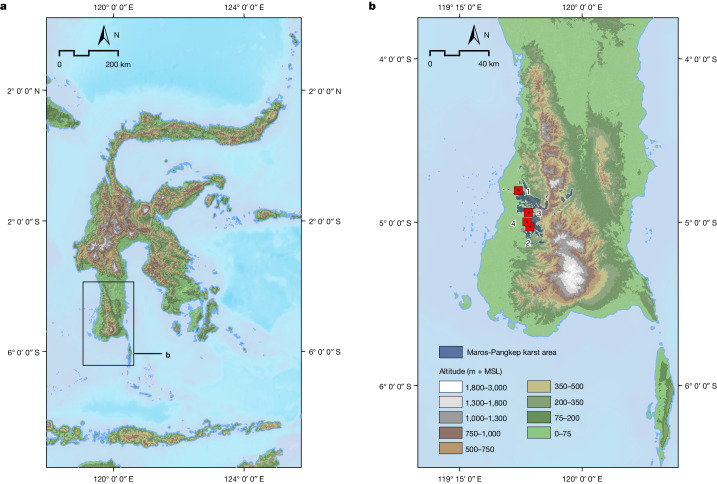
Map of the study area. **a**, The Indonesian island of Sulawesi, showing the location of the southwestern peninsula (area inside rectangle). **b**, South Sulawesi, with the limestone karst area of Maros-Pangkep indicated by blue shading. The locations of cave sites with dated Late Pleistocene rock art were as follows: 1, Leang Bulu’ Sipong 4; 2, Leang Karampuang; 3, Leang Tedongnge; 4, Leang Timpuseng. Map sources: GEBCO 2023 Grid; South Sulawesi karst database (Badan Lingkungan Hidup Daerah Provinsi Sulawesi Selatan; I. A. Ahmad, A. S. Hamzah). MSL, mean sea level.

Prehistoric rock art provides important insights into past human cultures, but is typically challenging to date in an accurate and reliable manner^4,5^. Over the past few decades, solution-based U-series methods have been used to produce early dates for rock art in several regions, including western Europe^6–9^, Island Southeast Asia^1–3,10,11^ and Russia^12^. In Spain, a hand stencil has been dated using solution U-series analysis of overlying calcite to at least 64.8 ka, and is therefore attributed to Neanderthals^9^; however, dating evidence presented for this image has been questioned^13–16^. Up until now, the earliest evidence for figurative art had comprised a naturalistic painting of a Sulawesi warty pig (*Sus celebensis*) at Leang Tedongnge in Maros-Pangkep (Fig. 1), which was dated using solution U-series to a minimum of 45.5 ka (ref. ^3^).

Thus far, only solution-based U-series methods have been used to date calcium carbonate deposits that formed in direct association with rock art. This approach involves the physical microexcavation of arbitrary layers of calcium carbonate and their chemical preparation before analysis using multicollector inductively coupled plasma mass spectrometry (MC-ICP-MS). The technique allows for small analytical error, but has some disadvantages for dating thin calcium carbonate layers associated with rock art, especially those with complex growth history; the latter includes the coralloid speleothems (also known as ‘cave popcorn’), which are most often found in association with rock art in limestone karst caves and shelters in Indonesia^1–3,11^. Here we used a laser-ablation U-series (LA-U-series) approach to date these thin calcite accretions in association with rock art. Instead of physically microexcavating arbitrary ‘spits’ using a rotary tool, as was done in the previous approach, the laser is focussed on a polished cross-section and requires no chemical preparation. The small spot size of the laser-ablation system (typically spot diameters of 44 μm) offers several advantages over solution-based methods: (1) it is fast and cost effective; (2) it requires a much smaller sample, and is therefore less destructive; (3) the spatial resolution obtained using this method allows the detailed growth history of the speleothem to be revealed and the age of the oldest deposit closest to the pigment layer to be precisely defined, improving accuracy; (4) it can be easily and rapidly demonstrated that the calcite has not undergone remobilization of uranium (and areas exhibiting these issues can be identified and avoided), which could be problematic for U-series dating (Methods, Supplementary Information and Extended Data Table 1).

Coralloid speleothems found in association with rock art often have complex internal morphologies that reflect their origin as aggregates of a cluster of cylindrical, mound-like calcite structures^17^, leaving overhanging features with gaps between older material that are infilled by carbonate materials of younger age. As the physical microexcavation procedure involves laterally collecting material from an arbitrary depth above the pigment layer—as opposed to sampling individual laminae—the resultant U-series age could, in some instances, be an average of the older mound material and the younger infill. Similarly, the lateral averaging of onion-like undulating layers can mix calcium carbonate material of different ages, sometimes resulting in a series of arbitrary subsamples with ages appearing not to be in chronological order. Such a complicated growth history may account for the small age reversals that are sometimes observed in previously dated samples using mechanical abrasion of arbitrary layers^2,3^. Here we used our LA-U-series approach to map the U-series isotopes across the surface areas of sample cross-sections. This approach enables us to understand the complex ways in which coralloid speleothems have formed, therefore enabling the analyst to identify, and avoid, small zones affected by diagenesis^18^.

The LA-U-series method generally provides age estimates with larger errors than the solution-based method, but it can result in genuinely older minimum ages for art as calcium carbonate material closer to the pigment layer can be analysed. This error can be improved by integrating a larger area of data; however, it can result in younger minimum ages as calcium carbonate material from later growth stages would need to be integrated. A more efficient way of minimizing this error involves slowing down the speed of the laser stage and increasing the integration time on the MC-ICP-MS, resulting in more datapoints for similar integrated areas. The trade-off is a substantial increase in the time needed to complete the analysis (Methods and Supplementary Information). We found that a spot size of 44 µm with a laser rastering speed of 21 µm s^−1^ (with a 2.097 s integration time) was optimal for most circumstances.

## New ages for previously dated art

**Fig. 2:**
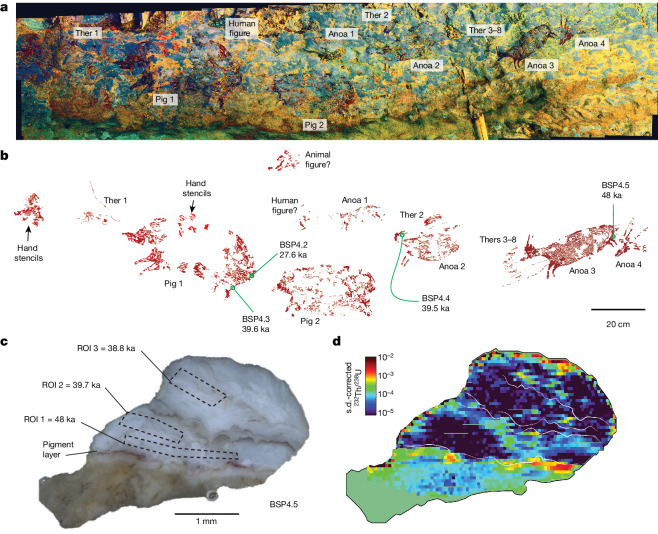
Dated rock art panel at Leang Bulu’ Sipong 4. **a**, Photostitched panorama of the rock art panel (using photographs enhanced using DStretch_Ire). Ther, therianthrope. **b**, Tracing of the dated rock art panel showing the results of LA-U-series dating. **c**, Transect view of the rock art sample BSP4.5 after removal from the artwork, highlighting the paint layer and the three integration zones (ROIs) and associated age calculations. **d**, LA-MC-ICP-MS imaging of the BSP4.5 ^232^Th/^238^U isotopic activity ratio.

To demonstrate the efficiency and reliability of this technique, we re-dated what was previously the oldest known surviving pictorial narrative, a rock art scene at Leang Bulu’ Sipong 4, which we had already dated to a minimum of 43.9 ka (ref. ^2^). At this cave site, a 4.5-m-wide panel on the rear wall comprises several figurative paintings of human-like figures interacting with Sulawesi warty pigs and dwarf bovids (anoas, *Bubalus* sp.) (Fig. 2). The former are depicted with material culture objects (spears and/or ropes) and some display what can be construed as attributes of non-human animals. These figures are interpreted as representations of therianthropes (composite human–animal beings)^2^. This enigmatic scene may represent a hunting narrative, while the prominent portrayal of therianthropic figures implies that the artwork reflects imaginative storytelling (for example, a myth)^2^.

We originally dated a total of four coralloid speleothems overlying animal figures on this panel to a minimum of 35.1 ka (35.7 ± 0.6 ka; sample BSP4.2), 43.9 ka (44.4 ± 0.5 ka; BSP4.3), 40.9 ka (41.1 ± 0.2 ka; BSP4.4) and 41 ka (41.3 ± 0.4 ka; BSP4.5). Using our LA-U-series method, those same speleothems and the associated artwork are now dated to a minimum of 27.6 ka (28.3 ± 0.6 ka; sample BSP4.2), 39.6 ka (43.2 ± 3.6 ka; sample BSP4.3), 39.5 ka (40.4 ± 0.9 ka; sample BSP4.4) and 48 ka (51.2 ± 2.2 ka; sample BSP4.5) (Fig. 2). Our LA-U-series approach provides either similar ages within error or older ages when compared with previous dates for the same samples (Fig. 2). The only exception is for sample BSP4.2, for which the LA-U-series data for the calcium carbonate deposits closer to the pigment layer are younger. We attribute this discrepancy to our selective avoidance of areas within the sample showing clear alteration (Methods, Extended Data Fig. 4 and Extended Data Table 1).

The rock art scene at Leang Bulu’ Sipong 4 can now be demonstrated to be at least 4,040 years older at 48 ka. On the basis of previous results, it appears that our mechanical microsampling approach^2^ either fortuitously avoided areas of diagenesis or that the averaging of microexcavated arbitrary layers rendered any localized digenesis inconsequential to the overall age calculation for these layers. Again the exception is for sample BSP4.2, for which the older minimum age for solution data near the pigment layer could be attributed to localized diagenesis. Our mapping data also show clear evidence of diagenesis occurring at the surface of samples and sometimes near tiny cavities within them. The latter areas would be impossible to avoid when microexcavating arbitrary spits for solution-based U-series methods and could result in erroneous age determinations. Using the LA-U-series mapping approach, these areas of localized digenesis can easily be avoided (that is, not integrated) when calculating U-series ages from map data. Notably, for example, the ^230^Th/^232^Th activity ratios for the laser-ablation regions of integration (ROIs) (Extended Data Table 1) are substantially higher than solution data for the same samples owing to the selective avoidance of areas with higher detrital content.

## Dating results for Leang Karampuang rock art

**Fig. 3:**
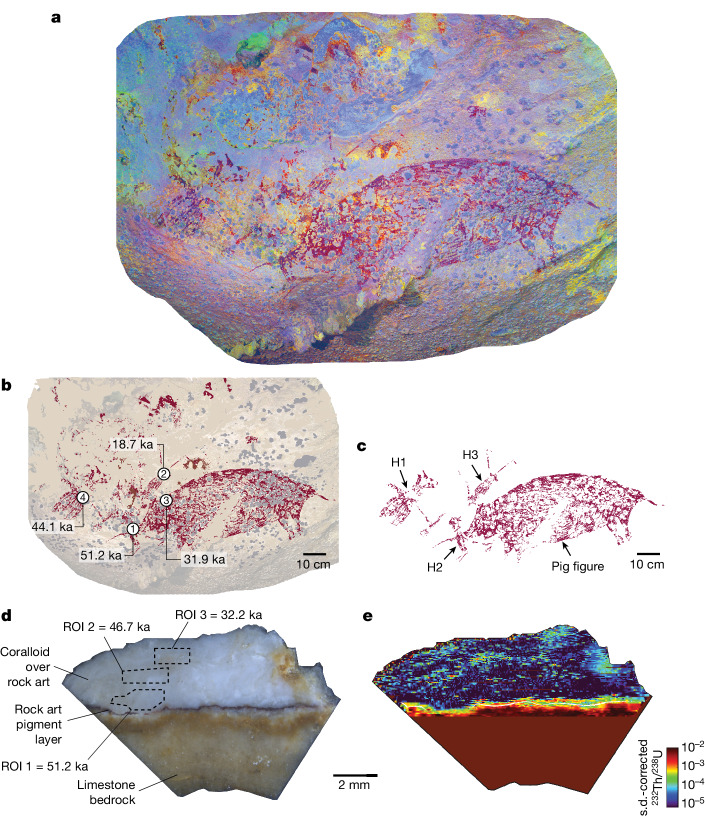
Dated rock art panel at Leang Karampuang. **a**, Photostitched panorama of the rock art panel (with photographs enhanced using DStretch_ac_lds_cb). **b**, Tracing of the rock art panel showing the results of LA-U-series dating. **c**, Tracing of the painted scene showing the human-like figures (H1, H2 and H3) interacting with the pig. **d**, Transect view of the coralloid speleothem, sample LK1, removed from the rock art panel, showing the paint layer and the three integration zones (ROIs), as well as the associated age calculations. **e**, LA-MC-ICP-MS imaging of the LK1 ^232^Th/^238^U isotopic activity ratio.

**Extended Data Fig. 1:**
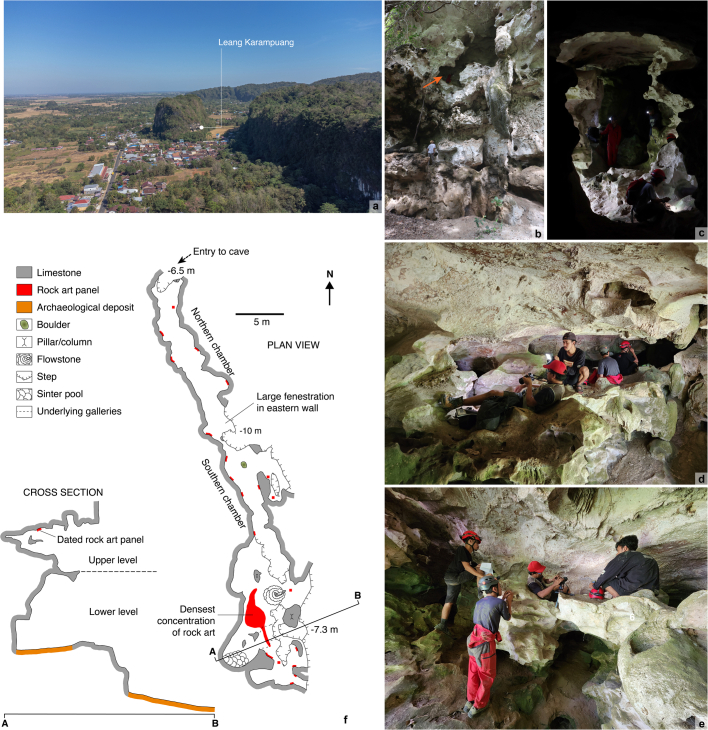
The Leang Karampuang rock art site in Maros, South Sulawesi. **a**, Aerial view of the limestone karst inselberg with Leang Karampuang at its base (the image was taken using a drone with a roughly southeast to northwest orientation); **b**, the upper level cave passage (indicated by orange arrow) containing the rock art at Leang Karampuang; pictured in this image is the large opening in the east wall of the passage that divides the cave into two chambers; **c**, northern chamber, looking from north to south; **d**, main rock art gallery in the southern chamber, containing the dated cave art panel – not clearly visible in the image are multiple paintings of pigs and other figures, and numerous hand stencils, on the overhead ceiling and adjoining upper walls; **e**, the dated rock art panel; **f**, plan view and cross-section of Leang Karampuang, showing the location of the dated rock art panel and other parietal artworks in the cave.

**Extended Data Fig. 2:**
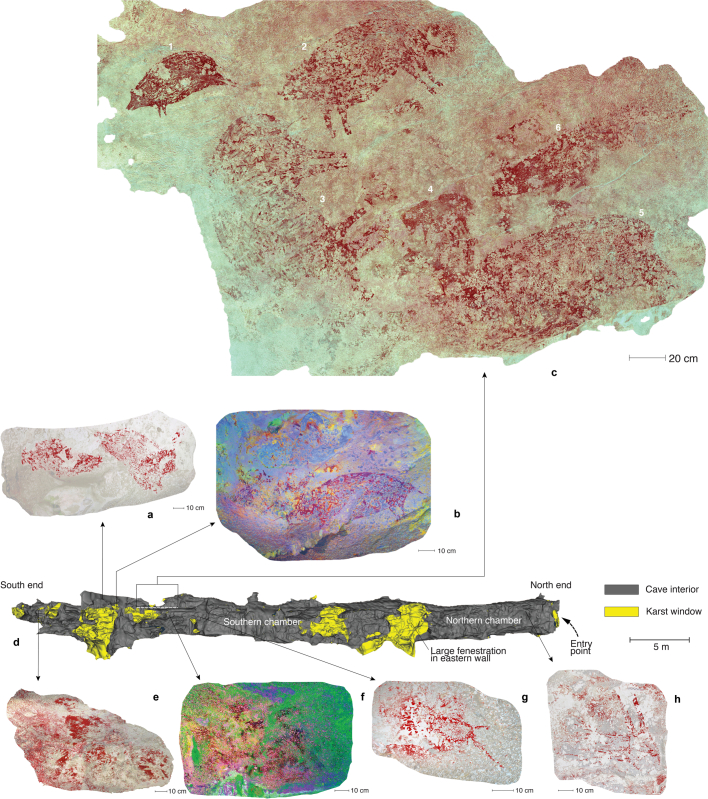
Key rock art panels at Leang Karampuang. Images of rock art panels are shown in **a**–**c** and **e**–**h**; **a**,**c**,**e**,**g**,**h** comprise digital tracings of rock art pigment overlying photographic representations of the underlying limestone rock face; **b** and **f** are photographs of rock art panels that have been enhanced using the DStretch_ac_lds_cb software. A 3D model of the cave passage containing the rock art gallery is presented in **d** (model produced using a Leica BLK360 Imaging Laser Scanner). **a**, Panel with three animal figures, probably pigs (the two figures on the right are in superimposition); **b**, the dated panel, a single composed scene depicting three human-like figures interacting with a Sulawesi warty pig (*S. celebensis*); **c**, a complex panel with six hand stencils and five representations of *S. celebensis* pigs (labelled 1–5), along with an unidentified animal figure (labelled 6); **e**,**f**, clusters of hand stencils, including some that are juxtaposed in such a way as to form what seem to be deliberate arrangements or compositions (a minimum of 10 and 11 individual hand stencils are identifiable in the panels shown in **e** and **f**, respectively); **g**, hand stencils and an image associated with an unidentified, animal-like figure; **h**, a possible representation of a dwarf bovid (anoa, *Bubalus* sp.), showing the head and neck area of the animal only.

Using the LA-U-series method, we also dated another rock art scene in Maros-Pangkep—one that again portrays human-like figures interacting with an animal (Figs. 1 and 3, Extended Data Figs. 1 and 2 and Supplementary Information). This ceiling panel was discovered in 2017 at the limestone cave of Leang Karampuang (Fig. 1). It is in a poor state of preservation owing to extensive exfoliation of the limestone rock surface, a process that has erased much of the art. The presence of abundant overlying coralloid growths (and other types of speleothems) further obscures the imagery (Fig. 3). The visible elements of the scene are dominated by a large (92 × 38 cm) naturalistic red painting of a suid (most probably *S. celebensis*). This animal figure is represented as a pictorial outline shown in side (profile) view with an infill pattern consisting of painted strokes or lines. It is therefore consistent in style with the visual convention used to represent pigs and other animals in the dated Late Pleistocene rock art of South Sulawesi, including at Leang Bulu’ Sipong 4 (ref. ^2^). Other pig motifs (*n* = 5) are present at Leang Karampuang, but do not seem to be associated with the dated panel (Extended Data Fig. 2 and Supplementary Information). In the latter, the pig is standing in a static position with its mouth partly open. At least three human-like figures (denoted H1 to H3) were depicted in close association with the pig as part of a single composition (Fig. 3). The former were portrayed using the same red-hued pigment and broadly the same stylistic convention as the pig, although they are smaller in size. At least two are arrayed in dynamic action poses near the head and face of the animal and seem to be engaged in some kind of close interaction with it. The largest human-like figure (H1, 42 × 27 cm) is represented with both arms extended; it has no legs, and it appears to be holding an item of material culture in its left hand, a rod-like object with a protuberance at both ends. The second human-like figure (H2, 28 × 25 cm) is positioned immediately in front of the pig with its head next to the snout. It also seems to have both arms extended and is holding a stick-like object of indeterminate form in its left hand, one end of which may be in contact with the pig’s throat area. The last human-like figure (H3, 35 × 5 cm) is depicted in an upside-down position with its legs facing up and splayed outwards. It also has its arms extended, with one hand reaching towards and seemingly touching the pig’s head. Pigment traces between H1 and H3 suggest that another figure may have originally been part of the scene. At least two hand stencils visible on the panel seem to be contemporary with the scene; another hand stencil, made using a darker pigment, is overlaid by the pig and was therefore produced earlier in time (Fig. 3). The actions taking place among the figures in this scene are difficult to interpret. In contrast to the dated artwork at Leang Bulu’ Sipong 4, this composition involving human-like figures and an animal does not seem to explicitly depict hunting activity, nor are therianthropes obviously represented, although we cannot rule out either.

**Extended Data Fig. 3:**
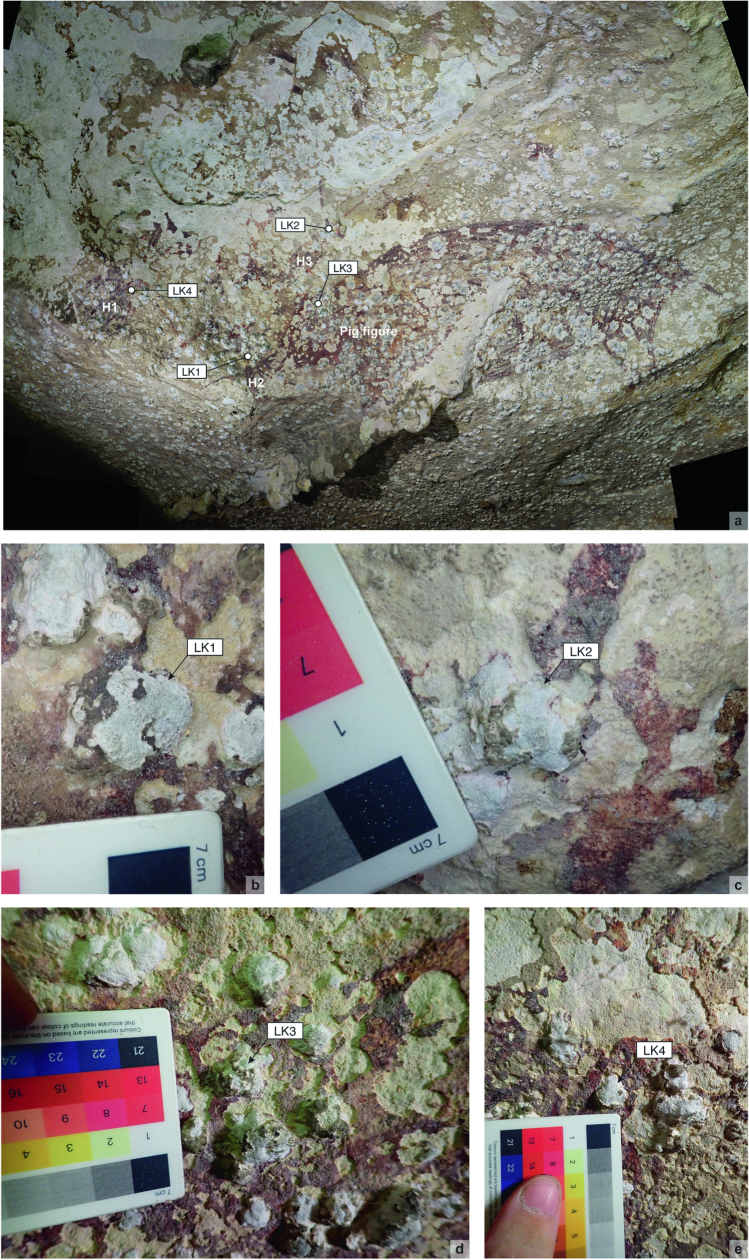
Coralloid speleothem samples from Leang Karampuang. **a**, Photograph of the dated rock art panel on the ceiling, a narrative composition or scene depicting at least four interacting figures: three are human-like in form (denoted H1-H3), and a fourth much larger figure is a naturalistic representation of a pig (denoted Pig figure) facing left. A coralloid speleothem was collected from each of the four figures (coralloid samples are denoted LK1 to LK4) and dated. Coralloid sample LK1 was collected from figure H2, which is positioned immediately in front of the snout of the pig figure and is holding a material culture object in its left hand, one end of which is positioned close to the throat area of the pig; LK2 was collected from H3, which seems to be depicted in an upside down position just above the head of the pig; LK3 was collected from the pig figure; and LK4 was collected from H1, a large anthropomorphic figure with extended arms and no legs depicted; as with H2, this figure is represented with a stick-like object in its left hand; **b**–**e**, photographs of each of the coralloid speleothems still in situ on the rock art panel prior to sampling: LK1 (**b**), LK2 (**c**), LK3 (**d**) and LK4 (**e**).

We collected four coralloid speleothems, one over each of the human-like figures and one over the closely associated pig motif (Fig. 3 and Extended Data Fig. 3). Samples LK1, LK2 and LK4 directly overlay H2, H3 and H1, respectively, while LK3 directly overlays the pig image. The results of LA-U-series dating of LK1 provided a minimum age of 51.2 ka (53.5 ± 2.3 ka), whereas the same method applied to LK2, LK3 and LK4 yielded minimum ages of 18.7 ka (19.2 ± 0.5 ka), 31.9 ka (34.1 ± 2.2 ka) and 44 ka (45.9 ± 1.9 ka), respectively (Fig. 3). Thus, we can demonstrate that this rock art scene was present at Leang Karampuang at least 51.2 ka, when the oldest dated coralloid (LK1) began to form on top of figure H2.

## Implications for the early history of art

At Leang Bulu’ Sipong 4, our LA-U-series dating work shows that the panel with the figurative art and composed scene is at least several thousand years older than previously established^2^, with a new minimum age of 48 ka. Furthermore, our use of the same method at Leang Karampuang shows that both forms of artistic expression (naturalism and narrative) date to at least 51.2 ka in the Maros-Pangkep karsts. It is evident from these findings that the use of figurative depiction has a particularly deep antiquity in the history of early human visual culture. Presently, the earliest widely accepted evidence for image-making by our species is from Middle Stone Age southern Africa (around 100–75 ka) and comprises geometric motifs (grid-like patterns) incised on small ochre nodules^19^. It is therefore an open question whether the origin of figurative depiction can be traced to an artistic culture that arose in Africa after the emergence of this early tradition of producing non-representational marks, or somewhere outside it after the dispersal of *H. sapiens*, including in Southeast Asia.

The new Sulawesi dates also challenge two key premises in the study of Pleistocene art, both of which are based on the rich record of artistic production in Upper Palaeolithic Europe (~40–10 ka): first, that the depiction of anthropomorphs or human-like figures did not become relatively common until towards the end of the Late Pleistocene^20,21^; and second, that the creation of obvious narrative compositions was rare or absent in early cave art generally^22–24^.

Concerning the latter, of the three oldest dated cave art panels now known from Sulawesi, Leang Karampuang (minimum age, 51.2 ka), Leang Bulu’ Sipong 4 (minimum age, 48 ka) and Leang Tedongnge (minimum age, 45.5 ka)^3^ all involve figures that are grouped together in such a way as to allow an observer to infer actions taking place among them. It is possible that the Leang Tedongnge art is older; however, we were unable to redate it using the new approach, as there was no calcium carbonate material remaining (the previous method involved the use of the entire sample)^3^. Two panels seem to comprise pictorial representations of human–animal relationships (Leang Karampuang and Leang Bulu’ Sipong 4), while the third depicts animals (*S. celebensis*) that are apparently interacting with each other^3^. Moreover, the panel at Leang Timpuseng (minimum age, 35.3 ka) portrays a pig standing on a painted line representing the land surface^1^, another convention used in scenic depiction^22–24^. The use of composed scenes in cave art may have enhanced the communicative potential of this visual medium^22–24^. In contrast to single-figure depictions, the agency of the juxtaposed figures constituting a narrative scene allows a story to be told through images in a manner that does not require the producer of the art to be present to convey the narrative to an audience^22–24^. Scene-making has therefore been linked to an increase in the potential for images that persisted on rock surfaces to transmit particular narratives (such as myths) over long periods of time, especially when combined with oral traditions^22–24^. On the basis of our dating work, it now seems that depictions of anthropomorphic figures (including therianthropes) interacting with animals appear in the Late Pleistocene cave art of Sulawesi at a frequency not seen elsewhere until tens of millennia later in Europe. This implies that a rich culture of storytelling developed at an early period in the long history of *H. sapiens* in this region — in particular, the use of scenic representation to tell visual stories about human–animal relationships.

## Methods

### Rationale for development and application of LA-U-series imaging

The use of the U-series disequilibrium method for rock art dating is relatively new and has drawn criticism from some authorities, especially in relation to the extremely old ages obtained for some European rock art and its attribution to Neanderthals^9,13–16^. In essence, the U-series disequilibrium method relies on the dating of calcium carbonate material present on the surface of rock art, providing a minimum age for the parietal imagery on which it formed. Four main principles need to be met for the valid use of this approach. (1) The associated motif needs to have an anthropomorphic origin; that is, there must be no doubt that the imagery associated with the calcium carbonate is an artwork produced by humans rather than some kind of natural marking. (2) The relationship between the rock art and the associated calcium carbonate must be unambiguous. (3) The dated samples must be relatively ‘pure’ (that is, the calcium carbonate must not be too contaminated by detrital material, which renders it ‘dirty’). (4) It needs to be empirically demonstrated that the dated calcium carbonate samples have remained a closed-system for uranium and thorium, and, especially, that there has been no ‘leaching’ of uranium over time, which could make the dated sample seem erroneously old.

The first principle is relatively straightforward to demonstrate in principle, especially when dealing with complex figurative artworks (for example, naturalistic paintings of animals), which are obviously cultural in origin. With regards to the second, it is not always a simple matter to demonstrate that a calcium carbonate sample of minuscule size collected for U-series dating completely overlies a rock art pigment layer or portion of, for example, an engraving. This is particularly the case when calcium carbonate powder samples are collected in situ using either a scalpel or a hand drill, and where researchers are prohibited from making direct physical contact with, and/or fully exposing, the pigment layer for closer inspection. This method usually results in a cone-shaped sample wherein only the apex or ‘pointy’ portion could be related to the underlying art^9^. As a result, some incorporated calcium carbonate powder could predate the motif^13^. It is sometimes also not clear whether the underlying surface assumed by the analyst to be the rock art has in fact been identified correctly as such—in some instances, it may be a portion of the underlying rock surface, rather than the adjacent artwork itself, that has been dated^9,13^. Concerning the third principle, the purity of the calcium carbonate sample can be easily and routinely assessed by measuring the ^230^Th/^232^Th activity ratios (see below). Finally, the presence or absence of closed-system conditions can be determined by measuring at least three subsamples; to demonstrate a closed system, these should either be of the same age within error or should get progressively younger towards the surface of the sample (see below). In this study, all of the measured ROIs fall within these categories.

Here we report an LA-U-series approach to dating rock art. This method enables us to more readily demonstrate the unambiguous relationship between the calcium carbonate material used for dating and the rock art pigment layer(s) to which it corresponds. Furthermore, the mapping of ^230^Th/^232^Th activity ratios across a sample cross-section enables the more accurate identification and selection of calcium carbonate material of the purest quality (that is, the part of the sample with the least evidence for contamination from detrital material), resulting in minor or insignificant age corrections. The latter outcome is impossible to achieve with solution-based methods, as these are based on a much less precise sampling approach that will typically incorporate both pure and dirty portions of a given mass of calcium carbonate. Uniquely, the mapping of U-series isotopes also enables the visualization of key areas or ‘zones’ within the sample in which calcium carbonate material has undergone mobilization of uranium or thorium. Once identified, these problem areas can then be deliberately avoided when selecting ROIs for dating. This is the main advantage of LA-U-series imaging compared with other U-series methods. It also enables the measurement of ROIs that are in clear chronological order, demonstrating closed-system conditions (see below). As has been previously indicated, the other main advantage of this approach to U-series dating is the ability to date calcium carbonate material that formed much closer to the rock art pigment layer. This is desirable, as dating the basal growth layers that accumulated directly on top of the surface of the artwork will potentially increase the minimum age of the associated anthropogenic imagery.

**Extended Data Fig. 4:**
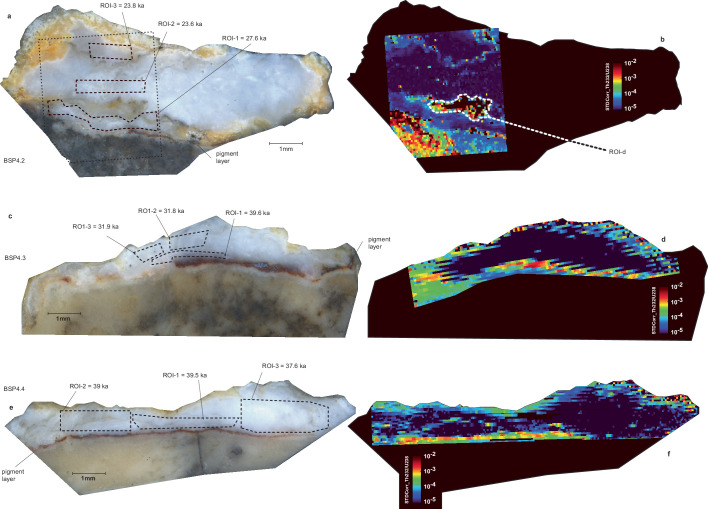
Transect views of three dated rock art samples from Leang Bulu’ Sipong 4. **a**, BSP4.2, **c**, BSP4.3, and **e**, BSP4.4. The pigment layer and the three integration zones (regions of integration, ROIs) are indicated, along with the associated age calculation for each sample. LA-MC-ICPMS imaging of **b**, BSP4.2, **d**, BSP4.3, and **f**, BSP4.4 ^232^Th/^238^U isotopic activity ratio. A zone of diagenesis is shown in **b** (ROI-d).

**Extended Data Fig. 5:**
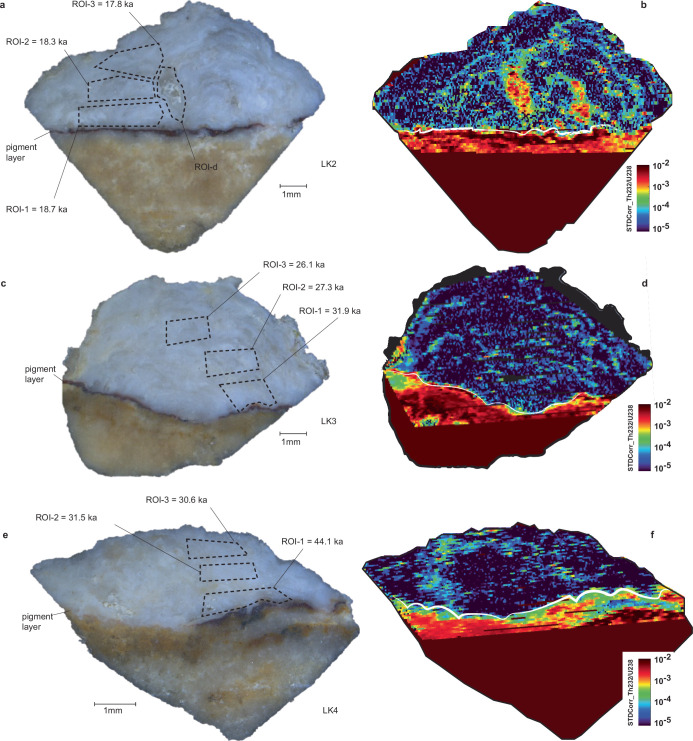
Transect views of three rock art samples from Leang Karampuang. **a**, LK2; **c**, LK3; and **e**, LK4. The pigment layer and the three integration zones (regions of integration, ROIs) are indicated, along with the associated age calculation for each sample. LA-MC-ICPMS imaging of **b**, LK2, **d**, LK3 and **f**, LK4 ^232^Th/^238^U isotopic activity ratio. A zone of diagenesis is also shown in **a** (ROI-d).

In cross-section, it is possible to visualize zones with discolouration at the surface of samples and sometimes within samples. These zones almost systematically correspond to a pronounced increase in the distribution of ^232^Th/^238^U. This ^232^Th/^238^U distribution is the easiest to visualize, but there is also usually a corresponding effect on other isotopes. For example, sample BSP4.2 displays a distinct brownish/blackish colour within an area of the sample (Extended Data Fig. 4). This zone corresponds to a marked increase in ^232^Th/^238^U and ^232^Th and a small increase in ^230^Th/^238^U (Extended Data Fig. 4 and Supplementary Fig. 5). This is attributed to the incorporation of detrital material. This incorporation of detrital material possibly occurred inside a porous area that could have been the subject of preferential uranium leaching. To illustrate this, an ROI (ROI-d) corresponding to this area was selected and an age of 38.4 ± 3.6 ka was obtained, thereby showing the effect of identified diagenesis on calculated ages (Extended Data Fig. 4 and Extended Data Table 1). This diagenetic zone is at least 7.2 ka older than ROI 1, corresponding to the layers immediately above the pigment layer and is identical to the solution age obtained for the same sample. The effect of diagenesis can also be seen clearly on LK2 sample sections. Visually, it is possible to identify a porous area in the middle of the sample (Extended Data Fig. 5). This area does not show a brownish-black colour as in sample BSP4.2, but corresponds to a significant increase in ^232^Th/^238^U, ^230^Th/^238^U and ^232^Th, as well as a significant decrease in ^238^U (Extended Data Fig. 5 and Supplementary Fig. 3). This porous area clearly underwent significant uranium loss and the incorporation of detrital material. To illustrate this, a new ROI (ROI-d) corresponding to this area was selected and an age of 28.5 ± 3.6 ka was obtained. This is at least 5.3 ka older in minimum age than the other ROIs for this sample. Again, this shows the effect of identified diagenesis on calculated ages (Extended Data Fig. 5 and Extended Data Table 1) and the efficacy of the LA-U-series imaging approach.

### Data measurement

A small segment (about 25–150 mm^2^) of each coralloid speleothem (*n* = 8) was removed from the rock art panels at Leang Bulu’ Sipong 4 (*n* = 4) and Leang Karampuang (*n* = 4) using a battery-operated rotary tool equipped with a diamond saw blade. Each speleothem sample was sawn in situ so as to produce a continuous microstratigraphic profile that extends from the outer surface of the speleothem through the pigment layer and into the underlying rock face. All of the sampled speleothems comprised multiple layers of visibly dense and non-porous calcite in clear association with painted motifs. In the laboratory, the remaining part of the samples from Leang Bulu’ Sipong 4 that were not microexcavated, and the new samples from Leang Karampuang, were sectioned along the growth axis with a diamond saw blade. The samples were then mounted in epoxy resin and polished at a 5 μm smoothness, exposing the pigment layers sandwiched between speleothem layers and/or bedrock.

The coralloid speleothem samples collected in this study formed by accumulation of thin films of water on cave surfaces over a long period of time. When precipitated from saturated solutions, and under ideal conditions, calcium carbonate usually contains small amounts of soluble uranium (^238^U and ^234^U), which eventually decay to ^230^Th. The latter is essentially insoluble in cave waters and will not precipitate with the calcium carbonate. This produces disequilibrium in the decay chain, a process in which not all isotopes in the series are decaying at the same rate. Subsequently, ^238^U and ^234^U decay to ^230^Th until secular equilibrium is reached. As the decay rates are known, the precise measurement of these isotopes enables the calculation of the age of the carbonate formation^25^.

All dating work was undertaken at the BIOMICS laboratory in the Geoarchaeology and Archaeometry Research Group (GARG) of Southern Cross University. U-series measurements were obtained using the ESI NW193 ArF excimer laser-ablation unit coupled to a MC-ICP-MS Thermo Fisher Scientific Neptune XT. Each sample was measured by a succession of parallel rasters across the exposed polished cross-section, enabling us to reconstruct an isotopic map of the precipitated calcite. Rasters had a different length to adapt to the irregular shape of the sample using the following parameters for mapping: a square spot size of 44 μm × 44 μm using the infinite aperture of the laser system matched by a translation speed of 21 μm s^−1^ and integration time of 2.097 s on the MC-ICP-MS Neptune XT. This combination of parameters enabled us to obtain a pixel within <0.1% deformation equivalent to a 44 μm × 44 μm datapoint (the exact translation speed to obtain an exact data-pixel of 44 μm × 44 μm would be 20.982 μm s^−1^). Other parameters for the data acquisition were as follows: 900 ml min^−1^ UHP He and 6 ml min^−1^ UHP nitrogen for the gas flow from the chamber to the ICP-MSs, frequency of 100 Hz for the laser frequency and an average of 1.74 J cm^−2^ sample fluence. ^234^U and ^230^Th were measured simultaneously, with uranium in the centre Faraday cup coupled with a secondary electron multiplier (SEM) and thorium on the L3 Faraday cup coupled to an ion counter (IC). All other Faraday cups were associated with a high-gain 10^11^ Ω amplifiers (the cup configuration was as follows: L3/IC(230); L2(232); L1(233); C/SEM(234); H1(235); H2(236); H3(238)). Baseline and drifts were corrected using NIST 610 and NIST 612 glass standards, while two corals (the MIS7 Faviid and MIS5 Porites corals from the Southern Cook Islands)^26^ were used to correct ^234^U/^238^U and ^230^Th/^238^U ratios and assess the accuracy of measurements (Supplementary Information). More information on data measurement is provided in the Supplementary Information.

### Image and data processing

Isotopic maps generated using LA-MC-ICP-MS data were produced using the Iolite software package^27^. Data were accumulated in a single file on the MC-ICP-MS Neptune XT system as follows: 5 min background, NIST610 (3×), NIST612 (3×), STD1 (3×), STD2 (3×), STD3 (3×), sample rasters (*n*×), STD3 (3×), STD2 (3×), STD1 (3×), NIST612 (3×), NIST610 (3×), 5 min background. For sample imaging sequences longer than 2 h, a set of standards (for example, STD1 (3×), STD2 (3×), STD3 (3×)) was incorporated in the middle of the measurement. Data reduction was performed using NISTs to assess drift and the 5 min background on each side of the measurements for baseline. One standard was used for correction of the isotopic ratios, while the other two were used as known values to check data accuracy (including for the matrix effect). Images were produced using a spectrum gradient colour distribution, with either a linear or logarithmic scale (specified for each sample on the isotopic maps). ROIs were carefully selected on the ^232^Th/^238^U, ^230^Th/^238^U isotopic ratio maps and U ppm maps to be as close to the pigment layer as possible, while avoiding diagenetic zones. Data errors were extracted and reported at 2 s.e. ROIs located immediately above the pigment layers were selected to calculate minimum ages relating to the underlying artworks. U-series data were integrated for individual ROIs, resulting in U-series ages and associated errors. Sufficient datapoints were also selected to minimize errors. The integration area of each ROI is reported in μm^2^ (Extended Data Table 1).

It is not uncommon for secondary calcium carbonate to be contaminated by detrital materials, such as wind-blown or waterborne sediments—a process that can lead to U-series ages that are erroneously older than the true age of the sample. This is due to pre-existing ^230^Th present in the detrital components. As the detrital/initial ^230^Th cannot be physically separated from the radiogenic ^230^Th for measurement, its contribution to the calculated ^230^Th age of the sample is often corrected for using an assumed ^230^Th/^232^Th activity ratio in the detrital component. Given that the detrital component within a cave is often composed of wind-blown or waterborne sediments that chemically approach the average continental crust, the mean bulk-Earth or upper continental crustal value of ^232^Th/^238^U = 3.8, corresponding to an ^230^Th/^232^Th activity ratio of 0.8—with an arbitrarily assigned uncertainty of 50%—has commonly been assumed for detrital/initial ^230^Th corrections^28^. In this regard, the degree of detrital contamination may be reflected by the measured ^230^Th/^232^Th activity ratio in a sample, with a higher value (such as >20) indicating a relatively small or insignificant effect on the calculated age and a lower value (<20) indicating that the correction on the age will be considerable^25^. As ^232^Th in the sample is largely present in the detrital fraction and plays no part in the decay chain of uranium, the detrital ^230^Th in a sample with a measured ^230^Th/^232^Th activity ratio of >20 would make up <0.8/20 (about 4.0% of the total ^230^Th in the sample), assuming that the mass fraction of ^232^Th from the detrital component is much larger than that from the authigenic component.

Sometimes, the assumed ^230^Th/^232^Th activity ratio of 0.8 (±50%) for the detrital component may not cover all situations. If the actual ^230^Th/^232^Th activity ratio in the detrital component substantially deviates from this assumed range, the detrital correction scheme may introduce considerable bias, especially to samples with a ^230^Th/^232^Th activity ratio of <20. In such situations, the ^230^Th/^232^Th activity ratio in the detrital component can be obtained through direct measurement of sediments associated with speleothems^9^, or computed using isochron methods or stratigraphic constraints^29^. In our case, our samples were relatively pure: the ^230^Th/^232^Th activity ratios of individual aliquots were extremely high. Corrections for detrital components were therefore calculated assuming the bulk-Earth values.

A conceivable problem with the U-series dating method is that calcium carbonate accretions can behave as an open system for uranium, in which the element can be leached out of the accretions or remobilized^30^. In such instances, the calculated ages will be too old because the dating method relies on the accurate measurement of uranium versus its decay product ^230^Th. In this study, this problem was tackled by avoiding porous samples and by measuring three aliquots (ROIs) from every sample. The ages of these subsamples were in chronological order or of similar ages within error, confirming the integrity of the dated coralloids. If uranium had leached out of the samples, a reverse age profile would have been evident (the ages would have gotten older towards the surface). Age calculations were performed using the UThwigl R package^31^ and compared to the IsoplotR (v.6.1) values^32^. Ages are reported with standard errors at the 2*σ* level.

### Reporting summary

Further information on research design is available in the Nature Portfolio Reporting Summary linked to this article.

## Online content

Any methods, additional references, Nature Portfolio reporting summaries, source data, extended data, supplementary information, acknowledgements, peer review information; details of author contributions and competing interests; and statements of data and code availability are available at 10.1038/s41586-024-07541-7.

## Supplementary information

Supplementary Information Supplementary Methods, Supplementary Figs. 1–6, Supplementary Tables 1 and 2 and Supplementary References.

Reporting Summary

## Figures and Tables

**Extended Data Table 1:**
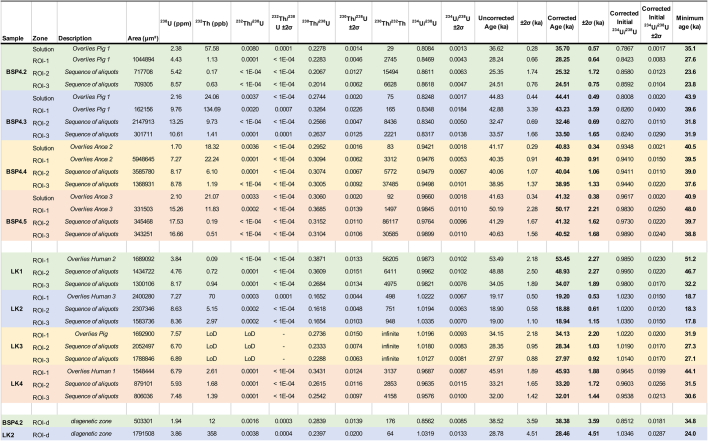
Uranium-series dating results for coralloid speleothem samples from Leang Bulu’ Sipong 4 and Leang Karampuang

This table contains the results of uranium-series disequilibrium dating calculated from three regions of integration (ROIs) for each coralloid speleothem sample (N = 8). These ROIs are in stratigraphic order and are all getting progressively younger towards the surface of the sample or are of the same age within error, demonstrating closed system conditions. Two additional ROIs (N = 2) show the effect of diagenesis (ROI-d) on a section of samples BSP4.2 and LK2. Solution measurements for BSP4 samples from immediately above the pigment layers^2^ (N = 4) are also provided for comparison. Note: ratios are activity ratios calculated from the atomic ratios. Errors are standard errors (SE) at 2σ level. Age calculations were performed using the UThwigl—R package^31^ and compared to the IsoplotR version 6.1^32^. Corrected ages were calculated assuming initial/detrital ^230^Th/^232^Th activity ratio equal 0.8 ( ± 100%) (the bulk-Earth value, which is the most commonly used for initial/detrital ^230^Th corrections). ^230^Th/^232^Th activity ratios are rounded up to zero decimal places and the errors are not included as they are below the decimal point. Uranium and thorium concentrations are approximations (qualitative) but can be used for comparisons between samples. LoD = limit of detection, denoting the lowest value that can be measured with statistical significance, as indicated in the table. Thorium concentration reaches the LoD at levels on the order of hundreds of parts per trillion (ppt), whereas the ^230^Th/^232^Th ratio is not statistically meaningful below 0.0001.

## Data Availability

The data supporting the findings of this study are provided in the Supplementary Information. Raw and additional Source data are available from publicly available Zenodo data repositories (10.5281/zenodo.10960856)^33^.
