# Supplementary material for: Narrative cave art in Indonesia by 51,200 years ago
Source: Nature. 2024 Jul 3;631(8022):814–8. doi: 10.1038/s41586-024-07541-7 (PMC11269172; doi:10.1038/s41586-024-07541-7)
Supplement: Supplementary file 1 — Supplementary Methods, Supplementary Figs. 1–6, Supplementary Tables 1 and 2 and Supplementary References. [file 41586_2024_7541_MOESM1_ESM.pdf]

nature portfolio

https://doi.org/10.1038/s41586-024-07541-7

Supplementary information

Narrative cave art in Indonesia by 51,200 years ago

In the format provided by the authors and unedited

Narrative cave art in Indonesia by 51,200 years ago

Adhi Agus Oktaviana[1,2*], **Renaud Joannes-Boyau**[3*], **Budianto Hakim**[4,5], **Basran Burhan**[5,6], **Ratno Sardi**[4,5] , **Shinatria Adhityatama**[1], **Hamrullah**[7], **Iwan Sumantri**[5,8], **M. Tang**[9], **Rustan Lebe**[5,10], **Imran Ilyas**[9], **Abdullah Abbas**[9], **Andi Jusdi**[5,9], **Dewangga Eka Mahardian**[2], **Sofwan Noerwidi**[2,5], **Marlon N.R. Ririmasse**[5,11], **Irfan Mahmud**[4,5], **Akin Duli**[5,8], **Laode M. Aksa**[9], **David McGahan**[6], **Pindi Setiawan**[12†], **Adam Brumm**[6*], **Maxime Aubert**[1,3,6*§].

[1] School of Humanities, Languages and Social Science, Griffith University; The Griffith Centre for Social and Cultural Research (GCSCR), Griffith University, Australia. [2] Pusat Riset Arkeometri, Organisasi Riset Arkeologi, Bahasa, dan Sastra, Badan Riset dan Inovasi Nasional, Jakarta; Center for Prehistory and Austronesian Studies (CPAS), Jakarta. [3] Geoarchaeology and Archaeometry Research Group, Southern Cross University, Lismore, NSW, Australia. [4] Pusat Riset Arkeologi Prasejarah dan Sejarah, Organisasi Riset Arkeologi, Bahasa, dan Sastra, Badan Riset dan Inovasi Nasional, Jakarta; [5]Pusat Kolaborasi Riset Arkeologi Sulawesi. [6] The Australian Research Centre for Human Evolution (ARCHE), Griffith University, Australia. [7] Korps Pecinta Alam, Universitas Hasanuddin, Makassar. [8] Departemen Arkeologi, Fakultas Ilmu Budaya, Universitas Hasanuddin, Makassar; [9] Balai Pelestarian Kebudayaan Wilayah XVIII, Makassar. [10] Badan Layanan Umum Museum dan Cagar Budaya, Direktorat Jenderal Kebudayaan, Jakarta. [11] Pusat Riset Arkeologi Lingkungan, Maritim, dan Budaya Berkelanjutan, Organisasi Riset Arkeologi, Bahasa, dan Sastra, Badan Riset dan Inovasi Nasional, Jakarta. [12] KK Desain Komunikasi Visual. Fakultas Seni Rupa dan Desain, Institute Teknologi Bandung.

[§] Corresponding author m.aubert@griffith.edu.au
* These authors contributed equally.
[†] Deceased

SI GUIDE

TABLE OF CONTENT

LIST OF SUPPLEMENTARY FIGURES .... page 2

Supplementary Figure 1: Comparison of MK16 values obtained by Laser Ablation and by solution analyses.. ……………………………………………………………......... page 2

Supplementary Figure 2: Comparison of MK10 values obtained by Laser Ablation and by solution analyses. ………………………..…………………………………………….. page 3

Supplementary Figure 3: LA-MC-ICPMS imaging of LK1 isotopic ratios………..page 4

Supplementary Figure 4: LA-MC-ICPMS imaging of BSP4.4 isotopic ratios…..… page 5

Supplementary Figure 5: LA-MC-ICPMS imaging of BSP4.4 isotopic ratios…..… page 6

Supplementary Figure 6: LA-MC-ICPMS imaging of BSP4.4 isotopic ratios…..… page 7

LIST OF SUPPLEMENTARY TABLES

Supplementary Table 1: Comparison of MK16 values obtained by Laser Ablation and by solution analyses (2σ SE). …………………………………………………..….. page 8

Supplementary Table 2: comparison of MK10 (only baseline and drift correction) values obtained by Laser Ablation and by solution analyses (2σ error)…………………….. page 9

SUPPLEMENTARY MATERIAL AND METHODS

Site description - Leang Karampuang ……………………………………………. page 10

Detailed methods …………………………………………………………...…….. page 11

REFERENCES………………………………………………………...…….. page 13

SUPPLEMENTARY FIGURES

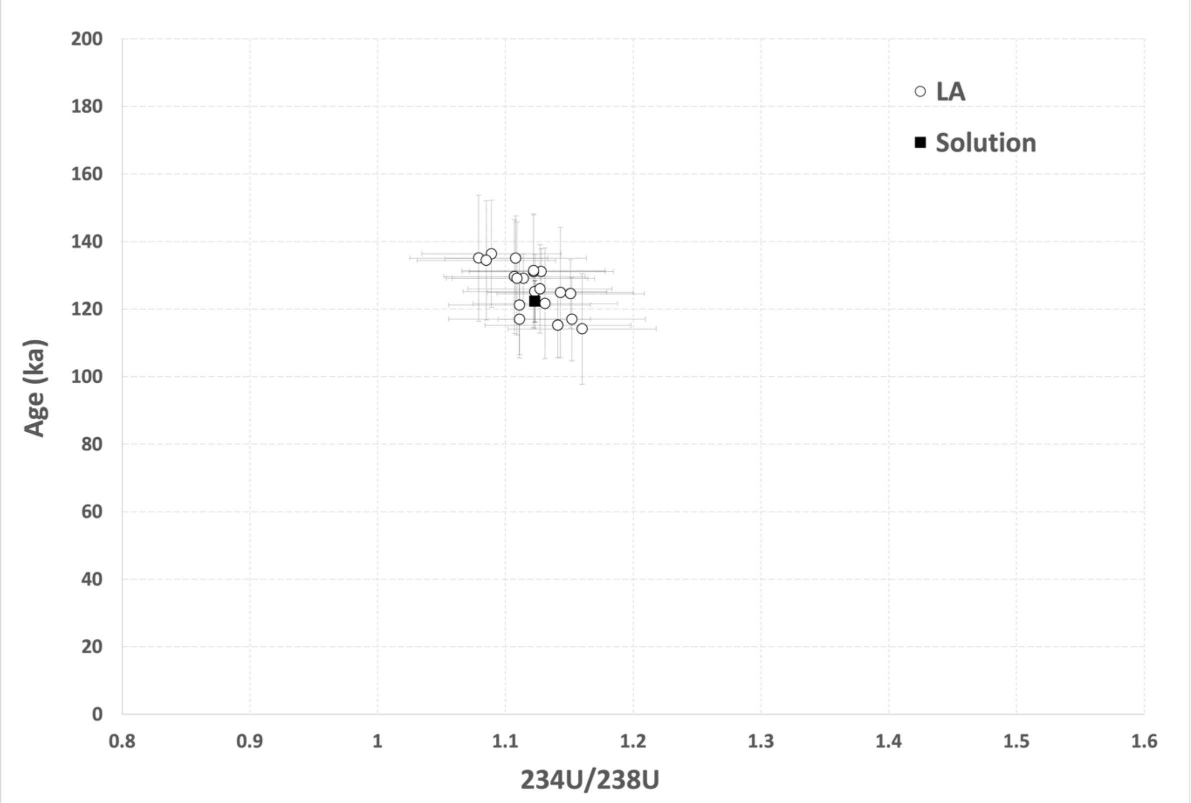

Supplementary Figure 1: Comparison of MK16 values obtained by Laser Ablation and by solution analyses. (error bars are 2σ SE; n=22 samples; refer to Supplementary table S1 for data values)

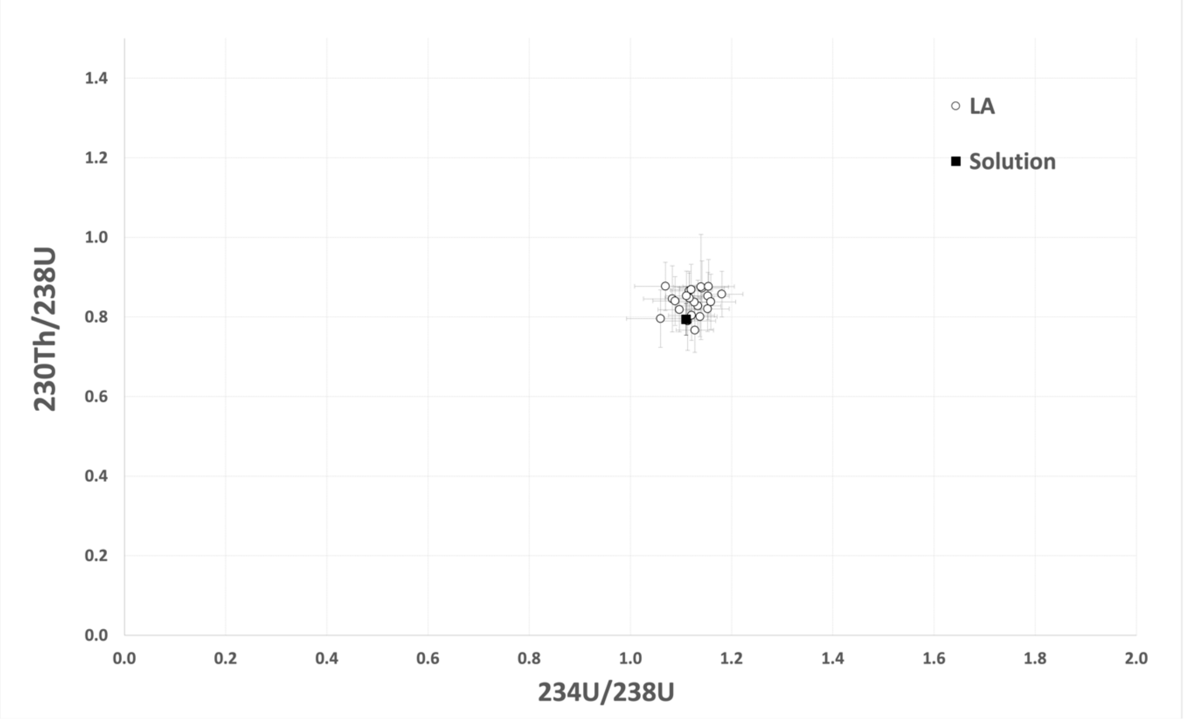

Supplementary Figure 2: Comparison of MK10 values obtained by Laser Ablation and by solution analyses (error bars are 2σ SE; n=26 samples; refer to Supplementary table S2 for data values).

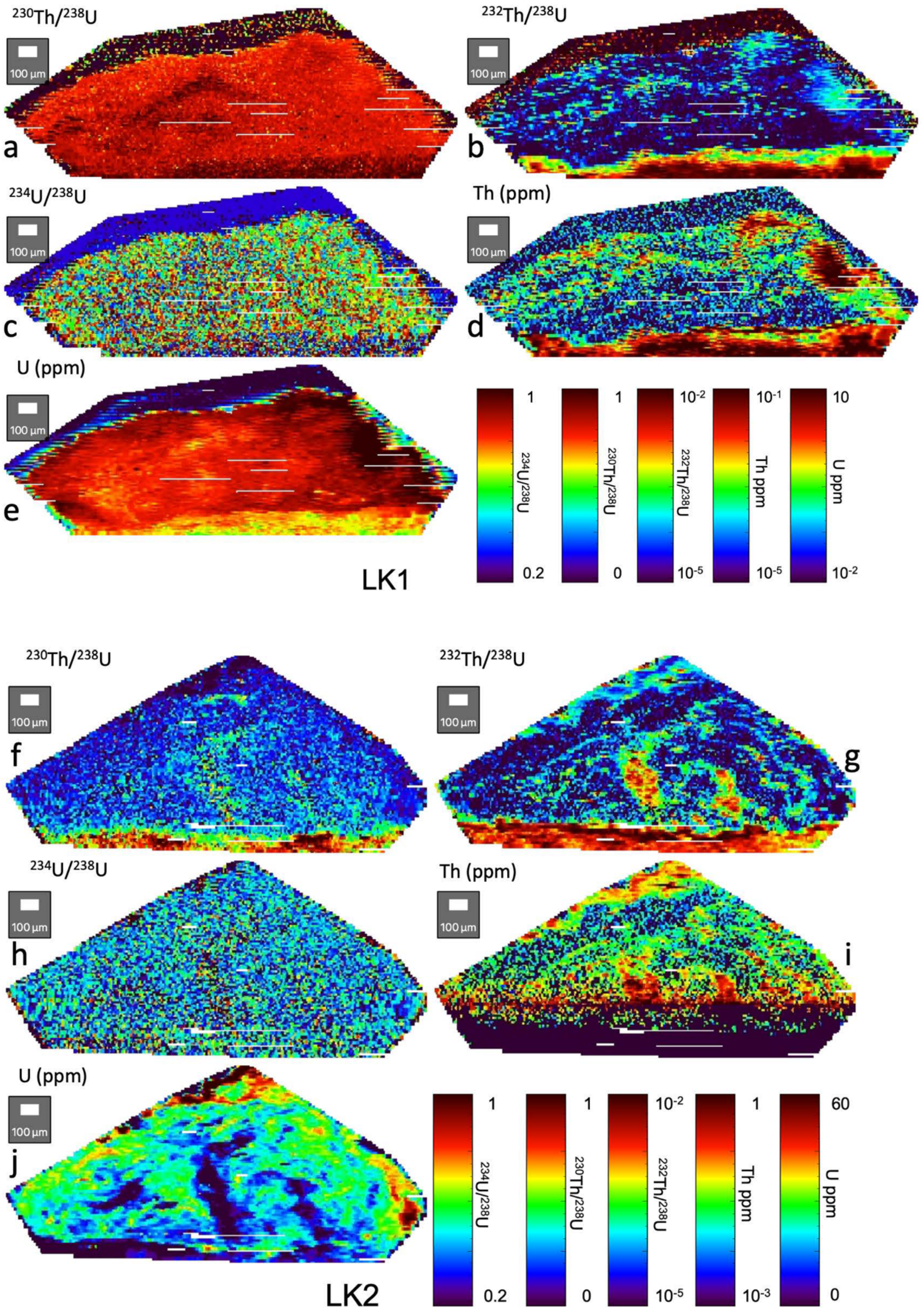

Supplementary Figure 3: LA-MC-ICPMS imaging of LK1 isotopic activity ratios. **a**, ^{230}Th/^{238}U; **b**, ^{232}Th/^{238}U; **c**, ^{234}U/^{238}U; **d**, ^{232}Th concentration (ppm) and **e**, ^{238}U concentration (ppm). And LA-MC-ICPMS imaging of LK2 isotopic activity ratios. **f**, ^{230}Th/^{238}U; **g**, ^{232}Th/^{238}U; **h**, ^{234}U/^{238}U; **i**, ^{232}Th concentration (ppm) and **j**, ^{238}U concentration (ppm).

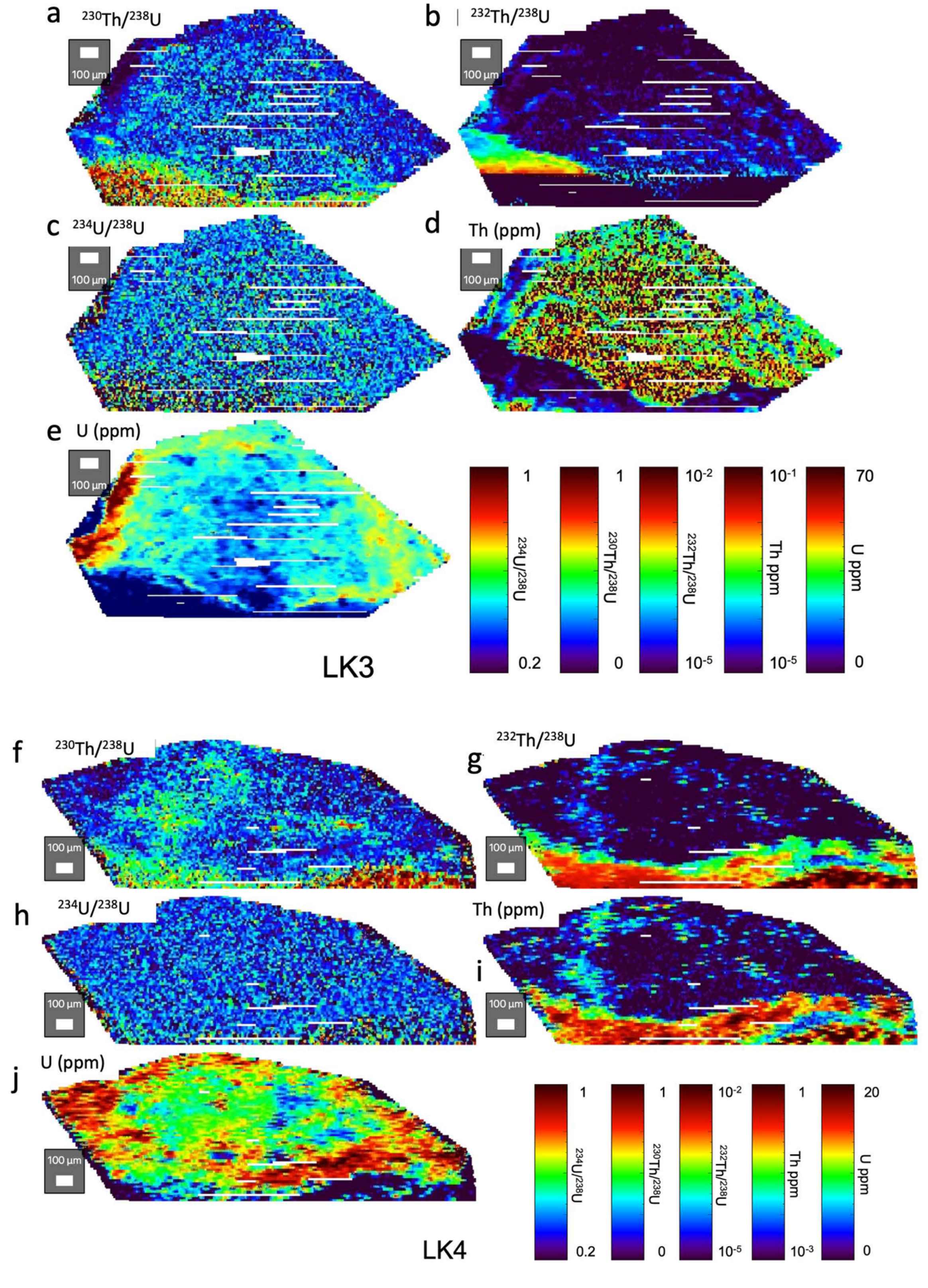

Supplementary Figure 4: LA-MC-ICPMS imaging of LK3 isotopic activity ratios. **a**, ^{230}Th/^{238}U; **b**, ^{232}Th/^{238}U; **c**, ^{234}U/^{238}U; **d**, ^{232}Th concentration (ppm) and **e**, ^{238}U concentration (ppm); and LA-MC-ICPMS imaging of LK4 isotopic activity ratios. **f**, ^{230}Th/^{238}U; **g**, ^{232}Th/^{238}U; **h**, ^{234}U/^{238}U; **i**, ^{232}Th concentration (ppm) and **j**, ^{238}U concentration (ppm).

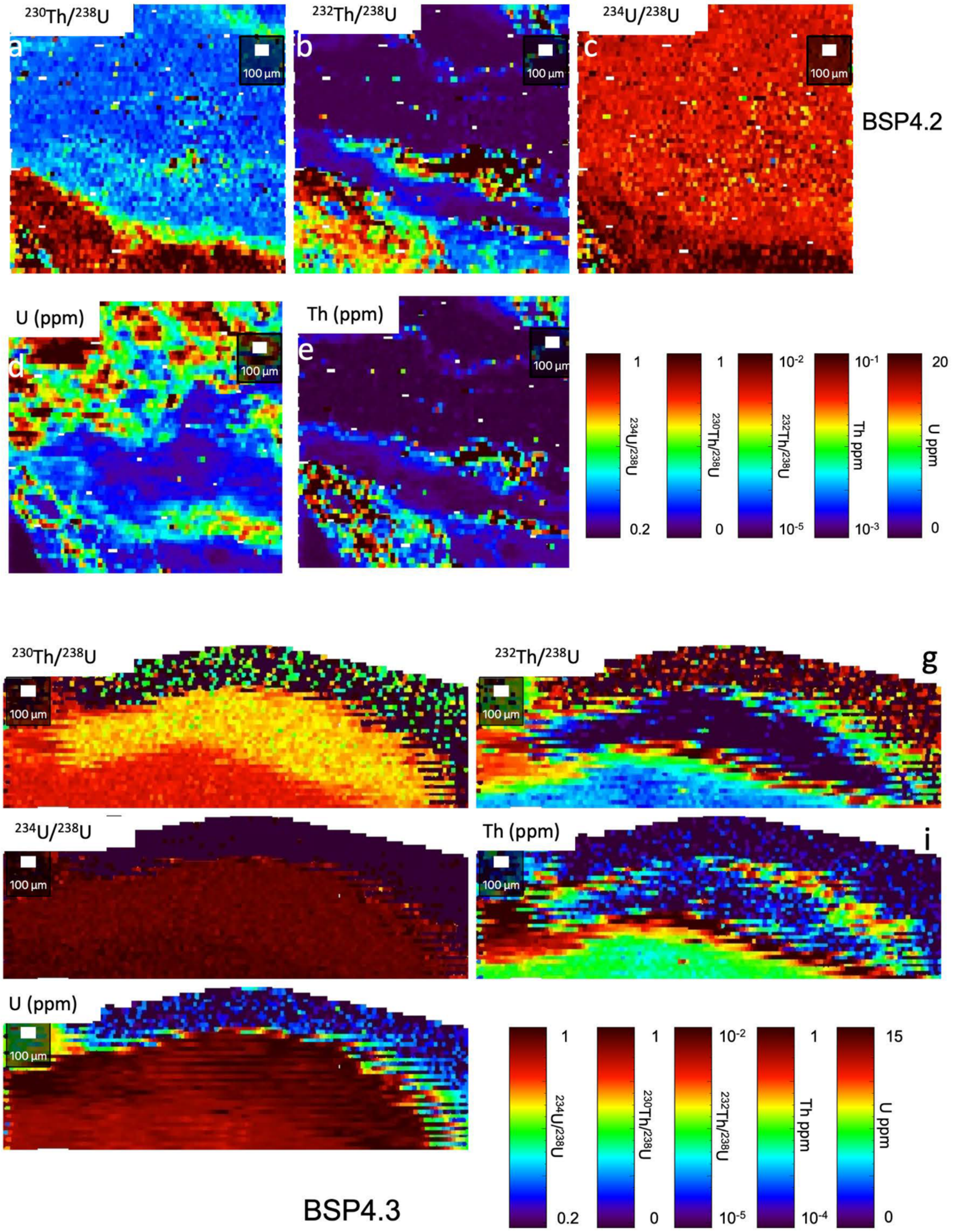

Supplementary Figure 5: LA-MC-ICPMS imaging of BSP4.2 isotopic activity ratios. **a**, ^{230}Th/^{238}U; **b**, ^{232}Th/^{238}U; **c**, ^{234}U/^{238}U; **d**, ^{232}Th concentration (ppm) and **e**, ^{238}U concentration (ppm) and LA-MC-ICPMS imaging of BSP4.3 isotopic activity ratios. **f**, ^{230}Th/^{238}U; **g**, ^{232}Th/^{238}U; **h**, ^{234}U/^{238}U; **i**, ^{232}Th concentration (ppm) and **j**, ^{238}U concentration (ppm).

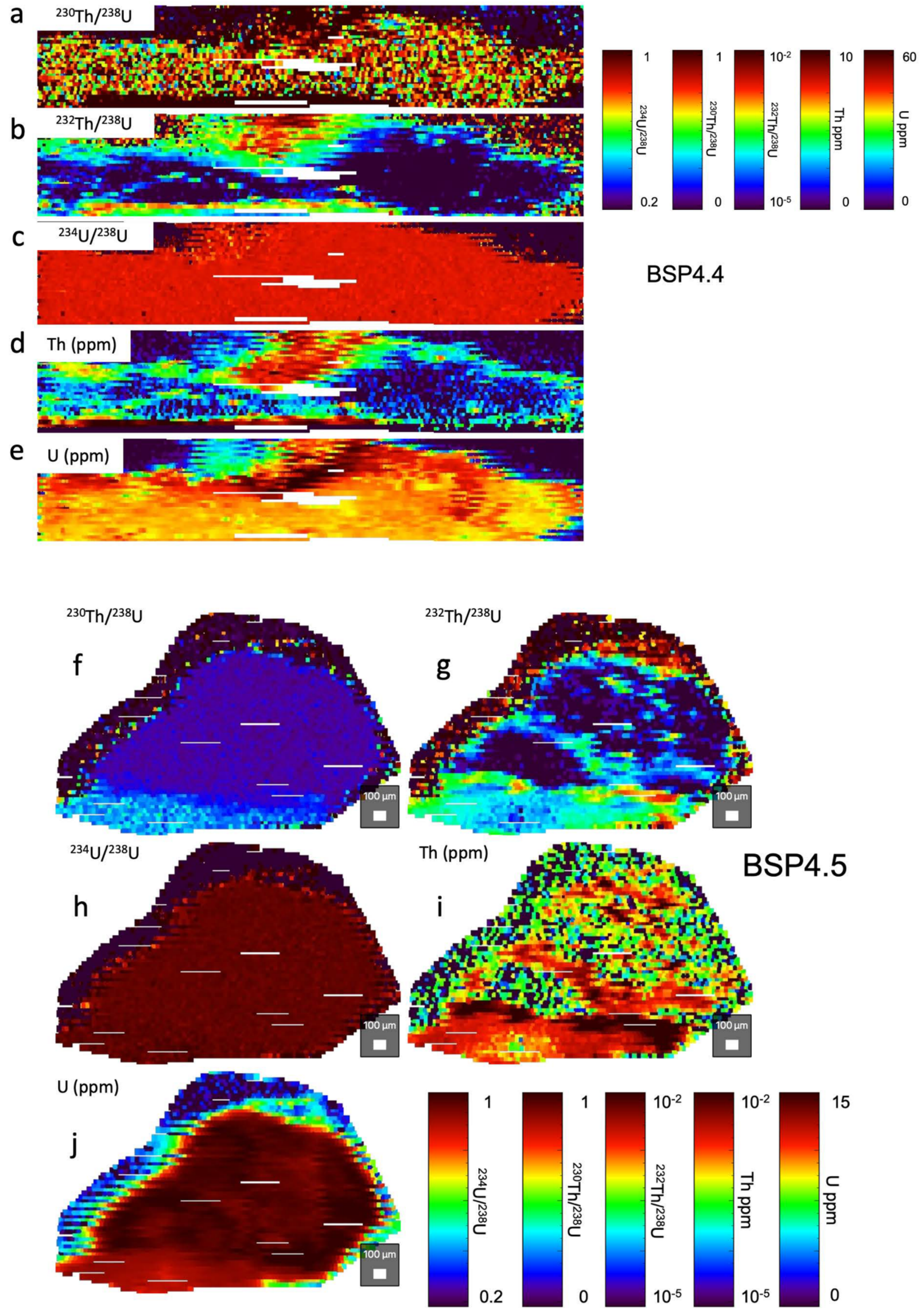

Supplementary Figure 6: LA-MC-ICPMS imaging of BSP4.4 isotopic activity ratios. **a**, $^{230}Th/^{238}U$; **b**, $^{232}Th/^{238}U$; **c**, $^{234}U/^{238}U$; **d**, ^{232}Th concentration (ppm) and **e**, ^{238}U concentration (ppm) and LA-MC-ICPMS imaging of BSP4.5 isotopic activity ratios. **f**, $^{230}Th/^{238}U$; **g**, $^{232}Th/^{238}U$; **h**, $^{234}U/^{238}U$; **i**, ^{232}Th concentration (ppm) and **j**, ^{238}U concentration (ppm).

SUPPLEMENTARY TABLES

MK16	*Area (μm^2)*	$^{230}Th/^{238}U$	error (2σ)	$^{234}U/^{238}U$	error (2σ)	T (ka)	error (ka) (2σ)
Solution		0.766	0.007	1.118	0.004	122.3	2.1
MK16_1	33000	0.774	0.064	1.131	0.044	121.6	16.4
MK16_2	33000	0.795	0.067	1.143	0.041	124.9	19.3
MK16_3	33000	0.786	0.055	1.114	0.053	129.1	7.3
MK16_4	33000	0.804	0.052	1.128	0.050	131.1	6.7
MK16_5	33000	0.800	0.050	1.151	0.040	124.5	10.2
MK16_6	33000	0.799	0.058	1.122	0.037	131.1	16.8
MK16_7	33000	0.782	0.057	1.107	0.036	129.6	16.9
MK16_8	33000	0.758	0.045	1.141	0.035	115.2	9.7
MK16_9	33000	0.757	0.048	1.111	0.028	121.2	14.8
MK16_10	33000	0.788	0.050	1.089	0.031	136.3	15.9
MK16_11	33000	0.742	0.050	1.111	0.038	117.0	11.5
MK16_12	33000	0.780	0.066	1.123	0.059	125.1	11.0
MK16_13	33000	0.776	0.059	1.079	0.037	135.1	18.7
MK16_14	33000	0.800	0.053	1.108	0.040	135.1	12.6
MK16_15	33000	0.786	0.049	1.127	0.033	126.0	13.1
MK16_16	33000	0.782	0.052	1.109	0.030	129.1	16.8
MK16_17	33000	0.800	0.050	1.151	0.040	124.5	10.2
MK16_18	33000	0.800	0.058	1.122	0.037	131.4	16.8
MK16_19	33000	0.779	0.057	1.085	0.036	134.4	17.7
MK16_20	33000	0.758	0.045	1.141	0.035	115.2	9.7
MK16_21	33000	0.773	0.080	1.152	0.074	117.0	12.4
MK16_22	33000	0.768	0.041	1.16	0.011	114.1	16.4

Supplementary Table 1: Comparison of MK16 values obtained by Laser Ablation and by solution analyses (2σ SE).

MK10	$^{230}Th/^{238}U$	error (2σ)	$^{234}U/^{238}U$	error (2σ)
Solution	0.794	0.014	1.110	0.004
MK10_1	0.845	0.083	1.083	0.057
MK10_2	0.872	0.069	1.141	0.044
MK10_3	0.828	0.065	1.133	0.045
MK10_4	0.852	0.059	1.153	0.042
MK10_5	0.818	0.056	1.097	0.042
MK10_6	0.869	0.064	1.120	0.042
MK10_7	0.838	0.069	1.159	0.049
MK10_8	0.804	0.063	1.121	0.046
MK10_9	0.857	0.057	1.181	0.041
MK10_10	0.801	0.050	1.137	0.034
MK10_11	0.875	0.132	1.139	0.054
MK10_12	0.837	0.043	1.126	0.031
MK10_13	0.840	0.062	1.088	0.044
MK10_14	0.866	0.049	1.116	0.036
MK10_15	0.848	0.063	1.116	0.048
MK10_16	0.853	0.063	1.111	0.041
MK10_17	0.820	0.057	1.152	0.043
MK10_18	0.767	0.055	1.127	0.037
MK10_19	0.876	0.068	1.154	0.051
MK10_20	0.790	0.074	1.113	0.055
MK10_21	0.818	0.056	1.097	0.042
MK10_22	0.869	0.064	1.120	0.042
MK10_23	0.838	0.069	1.159	0.049
MK10_24	0.804	0.063	1.121	0.046
MK10_25	0.877	0.061	1.069	0.061
MK10_26	0.796	0.072	1.059	0.067

Supplementary Table 2: Comparison of MK10 (only baseline and drift correction) values obtained by Laser Ablation and by solution analyses (2σ error).

SUPPLEMENTARY MATERIAL AND METHODS

Site description - Leang Karampuang

Leang Karampuang is a cave and rock-shelter complex located in the Simbang district of Maros-Pangkep. The site itself is situated at the southeastern foot of an isolated karst 'tower' (an outlier or inselberg) that is positioned 150 m south of a massive limestone hill mass. The tower has a perimeter of ~800 m and is surrounded by residential housing and cultivated rice-fields. Leang Karampuang has distinct lower and upper levels. The former constitutes a well-lit rock-shelter in which archaeological evidence for human occupation (e.g., stone artefacts, shells) abounds on the ground surface. The latter is a small cave passage positioned about 10 m above the main floor of the lower level. This cave is ~44 m-long and oriented approximately north-south. It is most easily accessed through a small opening located at the northernmost end at a height of around 6.5 m above the shelter floor. (It is also possible to reach the passage from the extreme southern end by climbing up a large fig tree). Approximately 25 m south of the entrance, the passage is dissected by a 5.5-m-wide section of floor collapse, resulting in a large fenestration in the eastern wall and a vertical drop of about 10 m to the lower level below. A 'scramble' across the western wall is required to span this gap and reach the southern side of the passage. Polished wear patterns on the limestone topography suggest that this area of floor collapse has been a feature of the passage since ancient times. (Pinch points, where foot traffic has been forced to follow a specific path, exhibit the same polished wear patterns). The fenestration divides the cave passage into northern and southern chambers. The former is dark to dimly lit, generally has a flat ceiling and is consistently around 2.5 m wide by 3.5 m high. The latter climbs gently from a ceiling height of ~3 m and the width is consistently around 3.6 m, except for a narrow (40-cm-wide) pass navigating large blocks of limestone wall collapse. Immediately south of this area, around 35 m from the main cave entrance, the floor simultaneously steps up and the ceiling drops down, creating a roughly 1.3-m-wide space. The remains of ceramic burial jars were identified in the cave passage. Each of the jars has been destroyed, presumably by looters, and their contents (including human skeletal remains) scattered across the ground surface. No other artefacts or archaeological features were visible on the cave floor in either chamber.

At each section of the passage illuminated by natural light, parietal artworks (mostly painted motifs and hand stencils) can be observed. (The only art-like markings identified in the lower level consist of non-representational arrangements of lines incised on limestone boulders and bedrock outcrops). The case-hardened surfaces of the walls and ceiling throughout the passage have undergone extensive exfoliation[1], probably over a long period of time, erasing much of the art. In some cases, colonies of coralloid speleothems also obscure the imagery. Despite this, some 161 distinct rock art motifs are discernible. This tally includes 128 hand stencils and 10 naturalistic paintings of animals, the majority of which (N = 6) are obvious depictions of suids – most likely the Sulawesi warty pig (*Sus celebensis*[2-3]) – as well as 13 images of animal-like forms. At least three figurative representations of anthropomorphs are evident, all located on a single panel (see below).

Most of the surviving rock art is concentrated on the overhead ceiling and adjoining west wall 'cornice panels' at the end of the southern chamber. This space is ~3.3 m wide (east-west) and ~7 m long (north-south), with a floor area consisting of an irregular surface of flowstone and limestone rock ledges (remnants of cave floor collapses) up to 3.5 m in height.

The roof height is also reduced to a distance of about 120-150 cm to less than a metre in this section, and to the southeast the ceiling drops down to almost completely close the chamber. Large openings in the passage's eastern side (karst 'windows' located between speleothem columns) allow daylight to enter the cave, illuminating the artworks.

Here, one prominent ceiling panel (Panel 4; located ~124 cm above the floor) features several paintings of suids (N = 5), a representation of an unidentified animal, and six hand stencils. The five suid figures (labelled 1-5 in Extended Data Fig. 2) were portrayed with markedly different body sizes and in various positions and orientations. These figures can all be interpreted as intentional portrayals of the endemic species *S. celebensis,* based on clear representations of head crests and other characteristic external morphological traits of this wild suid[2-3]. The unidentified animal (labelled 6 in Extended Data Fig. 2) has an elongate body, two short, paired assemblages that may represent limbs, and a row of strokes apparently representing hairs – it is possibly an incomplete figure of a pig. Below the smallest pig (labelled 4 in Extended Data Fig. 2), which is 39 cm long and 25 cm high, are two smaller figures (120 mm x 60 mm and 110 mm x 90 mm, respectively) that were painted with evident care but cannot be identified. At least one motif could represent a pig foetus or neonate.

Around 3 m to the south of this rock art panel with the five pig paintings is a separate panel located on the ceiling and adjoining cornice panel (the latter is oriented on a roughly 45° angle relative to the former). The highest point of this rock art panel is 116 cm above the floor and the lowest point is 87 cm above it. The panel artwork is dominated by a single composed scene (a narrative composition) executed in the same red to reddish-purple pigment. The scene consists of paintings of three human-like figures (denoted H1 to H3) interacting with a larger painted figure (length, 92 cm) that is a clear representation of a pig (most probably *S. celebensis*[2-3]). Two of the anthropomorphic figures (H1 and H2) seem to be holding items of material culture (stick-like objects) in their left hands. Traces of eight hand stencils are also discernible on the panel, with at least one produced using a dark brownish pigment; it is clearly superimposed by the scene. Traces of pigment indicate that other motifs were once present. These include portions of what appear to have been figurative motifs that based on their location were conceivably a part of the scene; however, the figures are not identifiable owing to the advanced state of exfoliation of the panel and the presence of numerous coralloid speleothems that have formed on the artwork (~30% of the pig figure motif is covered by these growths). A coralloid was collected from each of the four recognisable figures in the scene (H1-H3 and the pig figure), with Uranium-series analysis of these samples yielding the Late Pleistocene rock art ages reported in the present study.

Detailed methods

Tuning procedures were carried out using a NIST 610 glass standard with the following parameters: a 50 µm spot size, translation speed set to 5 µm per second, and a fluence of >1 J/cm^2 at the sample surface. The Multicollector Zoom Optic is optimized to ensure optimal peak shape for ^{238}U in faraday cup H3, with signal adjustments made to achieve a minimum of 1V for ^{238}U and maintain a 0.85 ratio factor between ^{232}Th and ^{238}U voltages (faraday cup L2 and H3, respectively). In the case of the samples analysed in this study, the average measures for ^{238}U exceeded 2V. Following tuning with NIST 610, rapid measurements were performed on both MK10 and MK16[5], with a particular focus on assessing the counts per

second (CPS) of uranium-234, and slightly adjusting parameters as necessary to optimise the signal. A spot size of 44 microns with a laser rastering speed of 21 microns per second with a 2.097 second integration time was optimal for most circumstances with the number of blocks varying depending on the measurement sequence. Yet, on average measurements were conducted with a total block number of ~10, with 200 cycles per block.

Background intensities (baseline) were subtracted from mean isotope signal intensities collected over a series of cycle (first and last 10 minutes and between every rasters for 30 seconds). Mean isotope signal intensities were also corrected for drift using the NIST612 standard at the beginning and end of every samples. The MK10 coral standard was used as a bracketing standard to correct for instrumental, elemental, and isotopic fractionation on the $^{234}U/^{238}U$ and $^{230}Th/^{238}U$ ratios. Corrected isotopic ratios were then used to calculated ages. The standard error for each isotope ratio, determined over n cycles, was analytically propagated through all stages of the standard corrections. Similarly, the baseline measurements and isotope ratios of the standards (both Laser and solution) were analytically incorporated into the final error of each isotope ratio. Finally, the errors were propagated through the age equation using a Monte Carlo technique, as described in the referenced publication on the UThwigl—R package[31] and IsoplotR[32].

Uranium and Thorium concentrations were obtained by comparing the background and drift corrected signal of the samples versus the background and drift corrected signal of the NIST612. Since there is no internal standard and these are two different matrices, these measurements are precise but may lack accuracy. However, these qualitative measurements are not used for isotopic ratios measurements and age calculations. They are useful when compared to other sub samples (ROIs) in order to identify uranium loss and detrital content.

Another coral sample, a MSI5 (MK16) Porite coral also from the Southern Cook Islands[5] is used to independently check the accuracy of the measurements and dating results. Both coral samples were reduced to powder to homogenise their values before being run by solution MC-ICPMS (Supplementary Table 1, 2, Supplementary Figure 1, 2). The remaining homogeneous powder samples were consolidated using a uranium- and thorium-free resin and used as standards for LA-MC-ICPMS. Standards were measured in the following order: 3x NIST610, 3x NIST612, 3x CoralSTD1 and 3x CoralSTD2 at the beginning of each sample and at the end in a mirroring order. The aforementioned list of standards was measured in between each sample to a maximum of 2 h in-between standard set.

Measurements for ^{230}Th and ^{234}U were conducted simultaneously with ^{234}U measured in IC1 with an SEM in the central axis position located behind an RPQ and the ^{230}Th on a separate faraday cup (L3) with the addition of an Ion Counter (IC or CCD). Cup configuration for all measurements was as follows: L3/IC(230); L2(232); L1(233); C/SEM(234); H1(235); H2(236); H3(238)).

$^{234}U/^{238}U$ and $^{230}Th/^{238}U$ isotopic ratios were corrected for elemental fractionation and Faraday cup/SEM yield by comparison with MK10 coral for which ratios were previously characterised internally by solution analysis (Supplementary Table 2, Supplementary Figure 2). Detrital-corrected ^{230}Th-U ages were calculated for each analysis using IsoPlotR[32] with an assumed detrital ($^{230}Th/^{232}Th$) activity ratio of 0.8 ± 0.8. Background subtraction, concentration quantification and ratio corrections were performed using Iolite™ software[27]. The corrected ($^{234}U/^{238}U$) and ($^{230}Th/^{238}U$) isotope ratios for the secondary standard (MK16

coral) were always within error of the value determined by solution analysis (Supplementary Table 1, Supplementary Figure 1).

Furthermore, we have previously published solution U-series data for all the BSP4 samples, and our new laser ablation U-series data closely aligns with these results. This demonstrates that using a coral calcium carbonate standard does not introduce any significant systematic bias. While our dates are reliable, it's important to note that they come with larger uncertainties, as the sensitivity of the laser ablation method cannot match the reproducibility of solution analyses.

The errors on isotopic ratios measurements for the coral standards (MK10 and MK16) are significantly larger than the samples ROIs. It is important to note that all reported errors are Standard Errors (SE), directly related to data population size. Individual MK16 2-SE errors are calculated using mean isotopic ratio values obtained over one raster measurement, representing approximately 33,000 μm^2. Comparatively, sample integration ROIs with similar superficies would yield analogous magnitudes of uncertainty. Our sample ROIs range in size from 162,156 to 5,948,645 μm^2 and therefore have a much larger population. This is why the 2-SE are much smaller.

References

1. Huntley, J., et al. The effects of climate change on the Pleistocene rock art of Sulawesi. *Sci. Rep.* **11**, 9833 (2021).

2. Brumm, A. et al. Oldest cave art found in Sulawesi. *Sci. Adv.* **7**, eabd4648 (2021).

3. Brumm, A. et al. Skeletal remains of a Pleistocene modern human (*Homo sapiens*) from Sulawesi. *Plos ONE* **16**(9), e0257273 (2021).

4. Aubert, M. et al. Earliest hunting scene in prehistoric art. *Nature* **576,** 442–444 (2019).

5. Woodroffe, C.D. et al. Stratigraphy and chronology of late pleistocene reefs in the Southern Cook Islands, south Pacific, *Quat. Res.* **35**, 246–263 (1991).
